# Effects of *Osmanthus fragrans* and *Sophora japonica* flower powders addition on hazardous compound formation, quality characteristics, and starch digestion in baked biscuits

**DOI:** 10.1016/j.fochx.2026.103915

**Published:** 2026-04-25

**Authors:** Yueliang Zhao, Xiangru Zhao, Daming Fan, Mingfu Wang, Hui Wang

**Affiliations:** a School of Public Health, Shanghai Jiao Tong University School of Medicine, Shanghai 200025, China; b College of Food Science and Technology, Shanghai Ocean University, Shanghai 201306, China; c School of Food Science and Technology, Jiangnan University, Wuxi, China; d Shenzhen Key Laboratory of Food Nutrition and Health, College of Chemistry and Environmental Engineering, Shenzhen University, Shenzhen 518060, PR China

**Keywords:** Biscuits, Edible flowers, Hazardous compounds, Physicochemical properties, Digestion

## Abstract

This study examined the impact of seven flower powders, including rose, peony, gardenia, chrysanthemum, honeysuckle, *Osmanthus fragrans*, and *Sophora japonica* flowers at 3% (*w*/w) on hazardous compound formation in baked biscuits. Among them, *Osmanthus fragrans* most strongly inhibited the formation of 5-hydroxymethylfurfural, acrylamide, and advanced glycation end products, followed by *Sophora japonica*. At addition levels of 1%–5%, both *Osmanthus fragrans* and *Sophora japonica* flower powders deepened biscuits color, reduced hardness, crispness, and chewiness, increased moisture content and water-holding capacity, and introduced a more porous microstructure. All formulations were sensorily acceptable. Digestibility tests showed that these two flower powders induced a shift from rapidly digestible to slowly digestible starch, accompanied by significant inhibition of α-amylase and α-glucosidase activities. This study provides theoretical and technical support for using edible flowers to enhance the safety, quality, and health-related properties of biscuits.

## Highlights

• Osmanthus flowers most effectively inhibited AA, 5-HMF and AGEs in biscuits.
• Flower powders improved biscuit texture, created porous structure and enhanced hydration.
• Flower powders reduced rapidly digestible starch and increased slowly digestible starch.
• Osmanthus flower biscuit extracts inhibited α-amylase and α-glucosidase.
• Edible flowers offer a dual-function strategy for safer, healthier bakery products.

## 1 Introduction

Edible flowers have been used for centuries in various cultures, including European and Asian cuisines, to enhance the sensory qualities (e. g. color, aroma, and flavor) and nutritional value of dishes, often consumed fresh in salads or processed into baked goods and beverages (Carboni et al., 2025). Beyond their visual appeal, edible flowers have recently attracted significant interest for their health-promoting properties, largely due to their richness in natural antioxidants such as vitamins and phenolic compounds (Carboni et al., 2025; Zhang et al., 2025). Among these, *Osmanthus fragrans* and *Sophora japonica* flowers stand out as traditional Asian ingredients, historically used in foods like osmanthus cakes and sophora porridge. Recognized as safe by the National Health Commission of China, their edibility is supported by long-term dietary use and toxicological evidence indicating no adverse effects at typical consumption levels. These flowers are notably rich in bioactive constituents, including phenolic compounds (e.g., chlorogenic acid, quercetin, rutin), dietary fiber, vitamins (C and E), and minerals such as calcium and iron (Liu et al., 2022; Wu et al., 2022). These components underpin their antioxidant, anti-diabetic, and anti-inflammatory activities (Carboni et al., 2025), highlighting their potential as functional ingredients for improving the nutritional and health profile of bakery products.

Biscuits are widely consumed globally, yet thermal processing induces the formation of contaminants including acrylamide (AA), 5-hydroxymethylfurfural (5-HMF), and advanced glycation end products (AGEs) via chemical reactions between ingredients (Teng et al., 2021; Zhang et al., 2023). AA, a probable human carcinogen (IARC Group 2 A), arises mainly from asparagine and reducing sugars during the Maillard reaction (Ma et al., 2022). Similarly, 5-HMF, another Maillard product, can be metabolized into 5-sulfoxymethylfurfural (SMF), which demonstrates mutagenic and genotoxic activities (Sahebi et al., 2025). Additionally, AGEs generated in this process are associated with diabetes progression and complications (Khan et al., 2020). Thus, suppressing the formation of these hazardous compounds in biscuits is essential to mitigate health risks.

The addition of natural antioxidants, such as phenolic compounds, fruit and plant extracts, has been demonstrated to mitigate hazardous compounds in bakery products. For example, seven polyphenols including punicalagin, ellagic acid, tyrosol, oleuropein, caffeic acid, chlorogenic acid, and epicatechin and three plant extracts (pomegranate peel, European cranberry bush juice, and olive mill wastewater) reduced AA formation in biscuits by 10.3% to 19.2% (Oral et al., 2014). Similarly, quercetin supplementation significantly lowers AGEs and 5-HMF in bread (Lin & Zhou, 2018; Zhang & An, 2017). Tara pod (*Caesalpinia spinosa*) and olive oil mill wastewater powder polyphenol extracts also exhibited strong inhibitory effects against 5-HMF formation, with suppression rates reaching 40–80% (Pedreschi et al., 2018; Troise et al., 2020). In another study, grape seed extract added to bread at 1200 and 2000 mg/kg reduced crust carboxymethyllysine (CML), a non-fluorescent AGEs, by over 30% and 50%, respectively (Peng et al., 2010; Zhang et al., 2023). Given that edible flowers are rich in natural antioxidants, we hypothesize that their incorporation could effectively inhibit the formation of hazardous compounds in bakery products.

The physicochemical properties of food, including color, texture, and firmness, significantly affect consumer acceptance, necessitating systematic evaluation. Studies indicate that plant-based ingredients can enhance food quality, for instance, 9% orange peel powder improves the appearance of gluten-free flatbread (Han et al., 2021), while 6% ginger powder increases bread hardness and reduces sensory scores (Balestra et al., 2011). Compared to these ingredients, edible flowers possess a distinct profile of natural pigments, phenolic compounds, and dietary fiber composition, which may lead to unique effects on biscuit quality. For example, flower-derived pigments and phenolics could modulate non-enzymatic browning by competing for reactive intermediates or scavenging free radicals, thereby influencing the color (Wu et al., 2024). Meanwhile, the soluble dietary fiber present in flowers can enhance water-retention capacity, improving texture (Kc et al., 2022). Nevertheless, systematic studies on the impact of edible flowers on the physicochemical properties of biscuits remain scarce. Furthermore, as a high-starch food, the digestibility of biscuits is closely linked to human health. Based on digestion rate, starch is classified into rapidly digestible (RDS), slowly digestible (SDS), and resistant starch (RS). RDS is digested within 20 min, causing a rapid rise in blood glucose, while SDS is slowly hydrolyzed over 20–120 min, aiding glycemic stability. RS escapes small intestinal digestion, functions as a prebiotic, and supports gastrointestinal health (Sivakamasundari et al., 2022). High SDS content promotes steady blood glucose levels, whereas high RDS may raise the glycemic index (GI), increasing risks of metabolic diseases. Long-term adherence to a low-GI diet is associated with reduced incidence of diabetes, heart disease, and certain cancers (Jenkins et al., 2024). However, whether edible flower addition influences starch digestibility in biscuits has not been systematically studied.

This study first systematically evaluated the inhibitory effects of seven edible flower powders (e. g. rose, peony, gardenia, chrysanthemum, honeysuckle, *Osmanthus fragrans*, and *Sophora japonica* flowers), each incorporated at 3% (*w*/w), on AA, 5-HMF, and AGEs formation in biscuits. Subsequently, the impact of their supplementation on the physicochemical properties, microstructure and sensory attributes of biscuits was investigated using colorimetry, texture analysis, and scanning electron microscopy. Finally, through in vitro digestion models and enzyme inhibition assays, the regulatory effects of edible flowers on starch digestibility and key digestive enzyme activities in biscuits were examined. This work provides a theoretical foundation and technical support for the utilization of edible flowers in enhancing the overall quality and health benefits of biscuits.

## 2 Materials and methods

### 2.1 Reagents and chemicals

Low-gluten flour, salt, edible oil, sugar, and edible flowers used for biscuit preparation were commercially procured, and ultrapure water was employed throughout the experiments. The chemical standards and reagents included acrylamide (AA, > 99% purity; DR Ehrenstorfer Gmbh, Germany), 5-hydroxymethylfurfural (5-HMF, ≥ 98% purity; Shanghai Yuanye Biotechnology, China), CML and d4-CML (TRC, Canada), chromatographic-grade methanol and acetonitrile, analytical-grade anhydrous ethanol and n-hexane (Shanghai McLean Biochemistry, China), as well as MCX solid-phase extraction columns (Shanghai Amperexperiment Technology, China). Enzymes and related reagents comprised pepsin (3000 U/mg; Shanghai McLean), α-amylase (3700 U/g; Beijing Solabao), trypsin and α-glucosidase (300,000 U/mL; Beijing Juming Biotechnology).

### 2.2 Preparation of edible flower powder

To prepare the edible flower powder, the petals of dried rose, peony, gardenia, chrysanthemum, honeysuckle, *Osmanthus fragrans*, and *Sophora japonica* flowers were separated from calyxes, ground into fine powder using a pulverizer, passed through a 200-mesh sieve, packaged in self-sealing bags, and stored at −20 °C until use.

### 2.3 Preparation of biscuits

Biscuits were prepared according to the method described by Hsiao et al. (2024) with modifications. The base formulation consisted of 7.5 g of low-gluten flour (containing 8.5 g/100 g crude protein, 76.4 g/100 g carbohydrate, and 1.1 g/100 g crude fat, as provided by the manufacturer), 0.1 g salt, 3.5 g sugar, 1.5 mL edible oil, and 1.5 mL purified water. Edible flower powder was added to the flour at substitution levels of 0, 1, 2, 3, 4, and 5% (*w*/w) and homogenized thoroughly. The dough was shaped using a 4.5 cm diameter circular mold and baked at 170 °C (upper heat) and 150 °C (lower heat) for 15 min in an oven (Hauswirt HO-30C, Qingdao, China). Finally, the baked biscuits were cooled, ground into powder, and stored at −20 °C pending analysis.

### 2.4 Determination of acrylamide (AA) and 5-hydroxymethylfurfural (5-HMF)

A modified method from Lemos et al. (2024) was employed to determine AA and 5-HMF levels. Briefly, ground biscuit samples (4.0 g) underwent a triple hexane wash (20 mL) for defatting, followed by air-drying. The defatted residue was then extracted with 20 mL methanol via 30-min ultrasonication using a KQ-500DE ultrasonic instrument (Kunshan Ultrasonic Instrument Co., Ltd., Kunshan, China). Following centrifugation (8000 ×*g*, 20 min) using a SIGMA 3-18 K centrifuge (SIGMA Laborzentrifugen GmbH, Osterode am Harz, Germany), the supernatant was subjected to clean-up by adding 0.7 mL each of Carrez's reagents I and II and subsequent centrifugation (8000 ×*g*, 25 min). The purified extract was rotary-evaporated to near-dryness at 40 °C, reconstituted in 2 mL ultrapure water, and filtered (0.22 μm) for HPLC analysis. HPLC separation was performed on a YMC-Pack ODS C18 column (4.6 × 250 mm, 5 μm), eluted isocratically with methanol-water (10:90, *v*/v) at 0.6 mL/min. The UV detector was set at 210 nm for AA (30 μL injection) and 284 nm for 5-HMF (10 μL injection). Analytes were identified by retention time and quantified by external standard calibration curves.

### 2.5 Determination of fluorescent AGEs

The fluorescent AGEs content was quantified following a procedure adapted from Jiang et al. (2024). Specifically, 1 g of the powdered sample was homogenized in 5 mL of ultrapure water and subjected to 20-min ultrasonic extraction using a KQ-500DE ultrasonic instrument (Kunshan Ultrasonic Instrument Co., Ltd., Kunshan, China). After centrifugation (10,000 r/min, 20 min) using a SIGMA 3-18 K centrifuge (SIGMA Laborzentrifugen GmbH, Osterode am Harz, Germany), the supernatant was collected, and the residual pellet was extracted twice more with 2 mL of ultrapure water per extraction. The combined supernatants were filtered through a 0.45 μm membrane. The fluorescence intensity of the final filtrate was recorded at respective excitation and emission wavelengths of 325 nm and 440 nm. The relative levels of fluorescent AGEs were expressed in arbitrary units (AU).

### 2.6 Determination of N^ε^-carboxymethyllysine (CML)

The CML analysis followed a modified protocol from Wang et al. (2024). Ground samples (1.0 g) were sequentially defatted with hexane (5 mL, three times), air-dried, and then reduced overnight at 4 °C using 1.5 mL of borate buffer (0.2 M, pH 9.2) and 1 mL of sodium borohydride solution (1 M in 0.01 M NaOH). Proteins were precipitated by adding trichloroacetic acid (TCA) to a final concentration of 20% (*w*/*v*), incubating at 4 °C for 2 h, and centrifuging. The resulting pellet was subjected to acid hydrolysis in 6 M HCl (5 mL) at 110 °C for 24 h. The hydrolysate was filtered, concentrated (10 mL), and derivatized. An aliquot (1 mL) was dried under nitrogen at 60 °C, reconstituted in 0.1 M HCl (2 mL), and spiked with CML-d4 internal standard (100 μL of 0.4 μg/mL). The solution was purified via a preconditioned MCX solid-phase extraction column, with elution using 5% ammonia in methanol. The eluate was evaporated, redissolved in 1 mL of ultrapure water, and 5 μL was injected for analysis. HPLC separation was achieved on a Shimadzu C18 column (2.1 × 100 mm, 3 μm), with a gradient of methanol (A) and 0.1% formic acid in water (B) at 0.2 mL/min: 0–0.1 min, 5% A; 0.1–7 min, 5% → 60% A; 7–9 min, 60% → 100% A; 9–10 min, 100% A; 10–15 min, re-equilibrated at 5% A. MS/MS detection was performed in ESI+ mode with MRM, monitoring the transition *m*/*z* 205 → 84 for CML and m/z 209 → 88 for the internal standard.

### 2.7 Determination of color of biscuits

The surface color of the biscuit samples was assessed using a CR-400 colorimeter (Konica Minolta, Japan). The L*, a*, b* color space parameters were recorded, where L* value measures lightness on a scale from 0 (black) to 100 (white), a* value signifies the red-green component (positive for red, negative for green), and b* value indicates the yellow-blue component (positive for yellow, negative for blue). The total color difference (ΔE) between the biscuits with added flower powder and the control group was calculated using the following equation: ΔE = [(L*-L_0_*)^2^ + (a*-a_0_*)^2^ + (b*-b_0_*)^2^]^1/2^, where L_0_*, a_0_*, and b_0_* represent the color parameters of the control biscuits, and L*, a*, and b* represent the color parameters of the biscuits with added flower powder. Additionally, the browning index (BI) for biscuits was calculated according to Djordjević et al. (2019). BI = [100 × (x - 0.31)] / 0.172, where x = (a* + 1.75 L*) / (5.645 L* + a* - 3.012b*).

### 2.8 Determination of texture of biscuits

The textural properties (hardness, crispness, and chewiness) of the baked and cooled biscuits were characterized using a TA-XT Plus texture analyzer (Stable Micro Systems Ltd., Godalming, UK) with calibrated 50 kg load cell. The instrument, equipped with a cylindrical P/50 probe, compressed each sample to 90% of its original height. The testing conditions were set as follows: pre-test, test, and post-test speeds of 2, 2, and 5 mm/s, respectively; a trigger force of 5.0 g; and a target strain of 1 mm. Measurements were conducted in quintuplicate, and the results are presented as mean values.

### 2.9 Scanning electron microscope (SEM)

The samples were carefully sectioned into thick slices and then defatted by soaking in petroleum ether for 5–8 h, with the solvent replaced every 2 h until no obvious yellow color was observed. Following defatting, the slices were freeze-dried to remove moisture. Finally, the defatted and dehydrated samples were coated with gold via ion sputtering under vacuum and observed using a scanning electron microscope (SEM; SU 1510, Hitachi Co. Ltd., Japan) at an accelerating voltage of 5 kV (Lu et al., 2022).

### 2.10 Moisture content and water holding capacity determination

Moisture content was determined according to the AOAC Official Method (AOAC, 2000) by drying samples at 105 °C to constant weight. It was calculated as the percentage of weight loss relative to the initial sample weight. Water-holding capacity (WHC) was measured in triplicate following Jia et al. (2020) with slight modifications. Briefly, 0.25 g of biscuit sample was mixed with 10 mL distilled water and equilibrated at 37 °C for 1 h. After centrifugation at 4800 r/min for 10 min, the sediment was weighed (wet weight, W_W_) and then dried to constant weight (dry weight, W_D_) in a forced-air oven at 110 °C. WHC was calculated as: WHC (g/g) = (W_W_ − W_D_)/ W_D_.

### 2.11 Sensory analysis

The sensory characteristics of the biscuit samples were assessed by 6 trained judgers using a 5-point hedonic scale (Zhao et al., 2019). The scale ranged from 1 (very poor) to 5 (excellent), with intermediate scores representing poor (2), fair (3), and good (4). Attributes evaluated included appearance, texture, taste, flavor, and overall acceptability. This sensory study did not require ethical approval per institutional guidelines. All participants provided informed consent prior to participation, having been fully informed of the study's purpose and procedures. Their rights and privacy were protected throughout the research: data were anonymized to safeguard identities, and participants retained the right to withdraw at any time without consequence.

### 2.12 Determination of rapidly digestible starch (RDS), slowly digestible starch (SDS), and resistant starch (RS)

An in vitro method (Lu et al., 2022) was employed to quantify RDS, SDS, and RS. Briefly, ground sample (0.5 g) was subjected to a simulated digestive process: oral digestion with α-amylase (50 U/mL in 0.2 mol/L nitrate buffer, pH 7.0, 15 s), followed by gastric digestion with pepsin (4 mg/mL in 2 mol/L HCl, 37 °C, 30 min). Following neutralization with 5 mL of 2 mol/L NaOH, intestinal digestion was carried out for 3 h at 37 °C using a pancreatic α-amylase/α-glucosidase mixture in phosphate buffer (0.2 mol/L, pH 6.0). Aliquots (125 μL) were withdrawn at 0, 20, 30, 60, 90, 120, and 180 min during the intestinal phase and the enzymatic reaction in each aliquot was immediately terminated by adding 5 mL of 85% ethanol. The glucose content was quantified using a commercial Glucose Assay Kit (Nanjing Yuandi Biotechnology Co., Ltd., China). RDS, SDS, and RS contents were calculated according to Eqs. (1), (2), (3), where G₀, G₂₀, and G₁₂₀ represent the glucose content (mg) at the respective hydrolysis times, and TS is the total starch content in the sample.

(1) $$\mathrm{RDS}\left(\%\right)=\frac{G_{20}-G_{0}}{\mathrm{TS}}\times 0.9\times 100$$

(2) $$\mathrm{SDS}\left(\%\right)=\frac{G_{120}-G_{20}}{\mathrm{TS}}\times 0.9\times 100$$

(3) $$\mathrm{RS}\left(\%\right)=100-\mathrm{RDS}\left(\%\right)-\mathrm{SDS}\left(\%\right)$$

### 2.13 Determination of estimated glycemic index (eGI) by hydrolysis kinetics

The eGI was derived from in vitro starch hydrolysis kinetics data. First, the hydrolysis percentage at time t (Cₜ) was modeled using a first-order kinetic equation (Eq. 4), where C∞ is the equilibrium concentration and k is the rate constant. The hydrolysis index (HI) was then calculated according to Eq. 5, where Gₜ is the glucose released at time t, and TS is the total starch content. Finally, the eGI was computed from the HI using the established conversion formula (Eq. 6).

(4) $$C_{t}=C_{\infty }\left(1-e^{-\mathrm{kt}}\right)$$

(5) $$\mathrm{HI}\left(\%\right)=\frac{G_{t}}{\mathrm{TS}}\times 0.9\times 100$$

(6) eGI=0.862×HI+8.198

### 2.14 α-amylase enzyme activity assay

The α-amylase inhibitory activity of the flower biscuit extracts (prepared as described in section 2.5) was evaluated using a modified 3,5-dinitrosalicylic acid (DNS) method (Visvanathan et al., 2021). Specifically, reaction mixtures containing flower biscuit extract (100 μL; 0.4–4.0 mg/mL in PBS) and amylose substrate (200 μL; 2.5 mg/mL) were pre-incubated at 37 °C for 10 min. The enzymatic reaction was initiated by adding 200 μL of α-amylase (1.25 U/mL) and continued for 10 min at 37 °C before being terminated by heat treatment (100 °C, 10 min). After cooling and centrifugation, the supernatant was purified via solid-phase extraction (Oasis MAX cartridges) to remove polyphenols that might react with DNS. The collected flow-through was reacted with DNS reagent, and the absorbance was measured at 540 nm. The inhibition percentage was calculated as follows:

(7) $$\text{Inhibition}\;\left(\%\right)=\frac{A_{\text{control}}-A_{\text{experiment}}}{A_{\text{control}}}\times 100\%$$

Where: A_control_ and A_experiment_ represent the absorbance of the control (with PBS replacing the extract) and sample groups, respectively.

### 2.15 α-glucosidase enzyme activity assay

α-Glucosidase activity was assessed according to Feng et al. (2022) with minor modifications. The assay mixture, containing 0.5 mL of α-glucosidase (10 U∙mL^−1^) and 0.5 mL of flower biscuit extract (1–8 mg/mL), was pre-incubated at 37 °C for 10 min. The reaction was then initiated by adding 1 mL of 5 mM *p*-nitrophenyl-α-D-glucopyranoside (pNPG) substrate and incubated for another 30 min at 37 °C. The reaction was quenched by adding 400 μL of 1 M Na₂CO₃, and the absorbance was measured at 400 nm. The inhibition rate was calculated as follows:

(8) $$\text{Inhibition}\;\left(\%\right)=\left(1-\frac{\left(A-B\right)-\left(C-D\right)}{A-B}\right)\times 100\%$$

Where A, B, C, and D represent the absorbances of the sample, sample control, control, and blank group, respectively.

### 2.16 Statistical analysis

All data are presented as the mean ± standard deviation (SD) of three independent replicates. Statistical analyses were conducted using SPSS (Version 21.0; SPSS Inc., Chicago, IL, USA). Differences among groups were assessed by one-way analysis of variance (ANOVA), followed by Duncan's post hoc test for multiple comparisons. A *p*-value of less than 0.05 was considered statistically significant.

## 3 Results and discussion

### 3.1 Effects of flower powder addition on the formation of hazardous compounds in biscuits

**Fig. 1:**
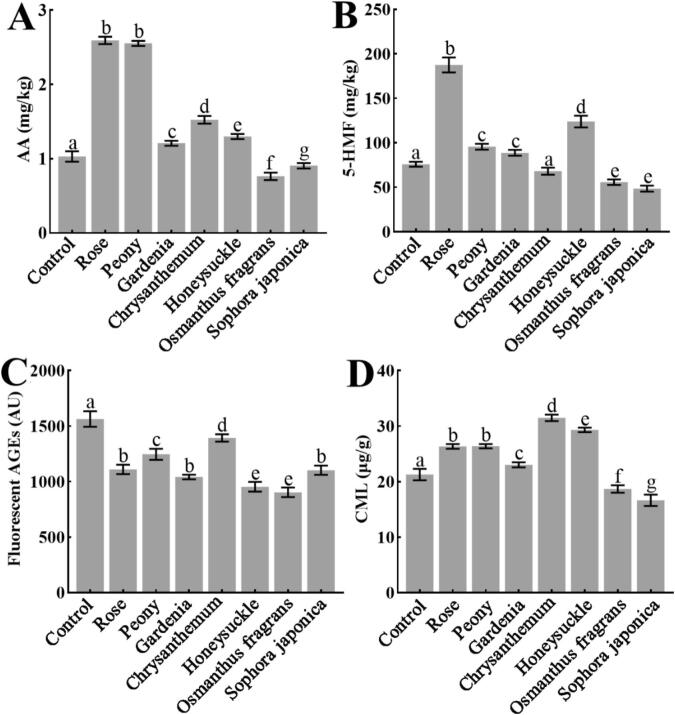
Effects rose, peony, gardenia, chrysanthemum, honeysuckle, *Osmanthus fragrans*, and *Sophora japonica* flowers (each added at 3%, *w*/w), on the formation of (A) AA, (B) 5-HMF, (C) fluorescent AGEs, and (D) CML in biscuits. *n* = 3. Bars with different letters (a-g) differ significantly (*P* < 0.05). (For interpretation of the references to color in this figure legend, the reader is referred to the web version of this article.)

The Maillard reaction, while imparting appealing color and flavor to biscuits, can also lead to the formation of potentially hazardous compounds such as AA, 5-HMF, and AGEs. This study examined the impact of seven edible flower powders, including rose, peony, gardenia, chrysanthemum, honeysuckle, *Osmanthus fragrans*, and *Sophora japonica* flowers, each incorporated at 3% (*w*/w), on the formation of these compounds (Fig. 1). Results indicated that *Osmanthus fragrans* flower most strongly inhibited the formation of AA and 5-HMF, reducing their levels by 25.97% and 33.7%, respectively. *Sophora japonica* flowers also showed significant inhibitory effects, with reductions of 12.14% for AA and 45.2% for 5-HMF. In contrast, rose and peony flower powders significantly promoted both AA and 5-HMF formation (Fig. 1A-B). All tested flowers inhibited the formation of fluorescent AGEs, with *Osmanthus fragrans* (45.1%) and honeysuckle (41.9%) flowers being the most effective. For N^ε^-carboxymethyllysine (CML), *Sophora japonica* flower showed the strongest inhibition (21.8%), followed by *Osmanthus fragrans* flower (12.2%), whereas the other five flowers promoted its formation.

**Fig. 2:**
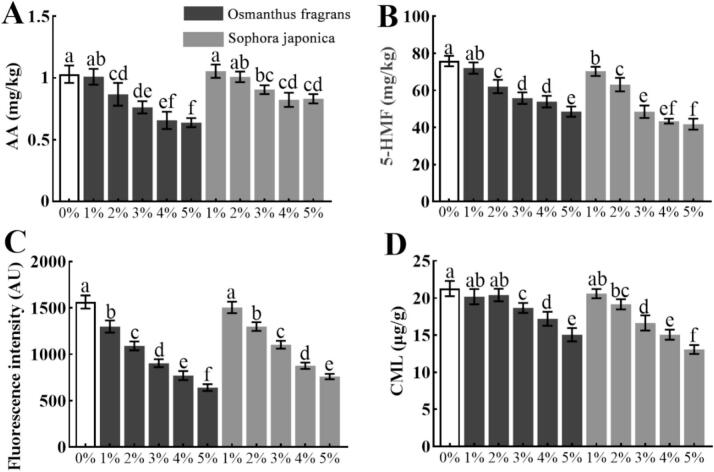
The formation of (A) AA, (B) 5-HMF, (C) fluorescent AGEs, and (D) CML in in biscuits with different addition levels of osmanthus fragrans and *sophora japonica* flowers. Bars with different letters (a-f) differ significantly (*P* < 0.05).

Given the strong inhibitory effects observed, *Osmanthus fragrans* and *Sophora japonica* flowers were further studied across a range of addition levels (1%–5%). As shown in Fig. 2A-B, *Osmanthus fragrans* flower suppressed AA and 5-HMF formation at all addition levels except 1%, exhibiting a dose-dependent effect. At 5% addition, the inhibition rates for AA and 5-HMF reached 38.00% and 35.99%, respectively. *Sophora japonica* flower also showed dose-dependent inhibition of 5-HMF, with a 44.92% reduction at 5% addition; however, significant AA inhibition was only observed at ≥3% addition.Both flowers also suppressed fluorescent AGEs in a dose-dependent manner (Fig. 2C), with *Osmanthus fragrans* showing stronger inhibition at each concentration. For example, at 5% addition, inhibition rates were 58.99% for *Osmanthus fragrans* and 51.38% for *Sophora japonica*. As for CML (Fig. 2D), both powders inhibited its formation dose-dependently, with inhibition rates of 29.22% (*Osmanthus fragrans*) and 38.64% (*Sophora japonica*) at the 5% level. Our results are consistent with previous studies showing that plant-based antioxidants can mitigate hazardous compounds in bakery products. For example, Aqueous extracts of clove at 4% reduced AA content in cookies by 50.9% (Zhu et al., 2011), while tara pod extract at 0.15% inhibited 5-HMF formation by 40% (Pedreschi et al., 2018). Grape seed extract at 0.2% reduced CML content in bread by 30% (Peng et al., 2010). Compared to these ingredients, *Osmanthus fragrans* and *Sophora japonica* flower powders exhibited comparable or stronger inhibitory effects on AA and 5-HMF, highlighting their potential as natural functional ingredients. The relatively weaker inhibition of CML by *Osmanthus fragrans* may be attributed to its higher lysine content, as lysine serves as a precursor for CML formation (Huang et al., 2022).

Given that polyphenols are known to mitigate Maillard reaction hazards through radical scavenging and trapping of reactive precursors and intermediates (Ou et al., 2019), the major phenolic compounds of the two selected flowers were characterized. Both *Osmanthus fragrans* and *Sophora japonica* were found to be rich in phenolic acids (e.g., chlorogenic, syringic, caffeic, protocatechuic, and *p*-hydroxybenzoic acids), flavonoids (e.g., quercetin), and polyphenol glycosides such as rutin, with also verbascoside identified in *Osmanthus fragrans* (Table S1), consistent with previous reports (He et al., 2016). These compounds have been shown to inhibit Maillard reaction by scavenging radicals and trapping α-dicarbonyls (Ou et al., 2019), which likely contributes to the observed suppression of hazardous compound formation.

### 3.2 Effects of flower powder addition on the physicochemical properties, microstructure and sensory attributes of biscuits

The physicochemical properties and microstructure of food directly influence its sensory quality and eating experience. This study evaluated biscuit characteristics in terms of physical characteristics (diameter, thickness, and spread ratio), color, texture, microstructure, and moisture properties. Table 1, Table 2 present the physical properties of biscuits fortified with *Osmanthus fragrans* or *Sophora japonica* flower powder. The fortified samples showed a decreasing trend in diameter and thickness compared to the control, though the differences were not statistically significant. This trend can be attributed to the lower hygroscopicity of wheat flour relative to dried plant powders, which allows more water to remain available for gluten network development and supports biscuit height (Ramashia et al., 2021). A similar trend has been reported for biscuits enriched with roselle calyx powder (Hernández-Nava et al., 2023). The spread ratio, a key quality attribute related to texture and mouthfeel, tended to increase in fortified samples, but again without significant difference from the control. A higher spread ratio is generally desirable in biscuits (Kohli et al., 2023). Together, these results suggest that the incorporation of edible flower powders does not adversely affect the physical properties of biscuits and may even contribute to quality attributes favored by consumers.

**Table 1:** Impact of *Osmanthus fragrans* flower powder addition (1%–5%) on the color, texture, physical properties, moisture content and water retention of biscuits.

Parameters	Control	*Osmanthus fragrans* flower powder				
		1%	2%	3%	4%	5%
Color						
L^⁎^	49.19 ± 0.89^a^	48.20 ± 0.58^b^	46.82 ± 0.49^c^	44.43 ± 0.57^e^	42.28 ± 0.49^fg^	40.63 ± 0.48^h^
a^⁎^	8.16 ± 0.22^a^	7.43 ± 0.15^bc^	7.32 ± 0.19^c^	7.21 ± 0.17^cd^	6.90 ± 0.15^e^	6.54 ± 0.33^f^
b^⁎^	27.17 ± 0.38^a^	26.03 ± 0.24^bc^	25.61 ± 0.21^c^	23.31 ± 0.43^de^	22.29 ± 0.42^f^	21.71 ± 0.42^g^
ΔE	–	23.87 ± 0.33^a^	24.18 ± 0.23^a^	25.65 ± 0.38^b^	26.97 ± 0.47^c^	28.32 ± 0.34^d^
BI	88.35 ± 2.47^a^	85.09 ± 2.17^b^	86.59 ± 1.55^ab^	82.79 ± 1.78^d^	83.33 ± 2.38^c^	84.51 ± 1.66^bc^
Hardness (g)	24,360.35 ± 586.33^a^	21,149.66 ± 884.48^b^	18,235.62 ± 682.51^c^	20,879.32 ± 658.12^b^	16,646.20 ± 565.89^d^	14,574.50 ± 610.28^e^
Crispness (g)	7376.29 ± 269.75^b^	7258.89 ± 222.68^bc^	6668.63 ± 127.10^d^	7584.70 ± 99.14^a^	7075.86 ± 72.79^c^	6691.59 ± 124.35^d^
Chewability (g∙cm)	8012.22 ± 308.66^a^	7362.03 ± 208.69^bc^	6927.18 ± 191.38^d^	7388.85 ± 223.06^bc^	6938.35 ± 261.69^d^	6024.21 ± 259.20^e^
Diameter (mm)	46.30 ± 1.21^a^	46.14 ± 1.17^a^	46.27 ± 1.26^a^	46.28 ± 1.19^a^	46.14 ± 1.32^a^	46.19 ± 1.35^a^
Thickness (mm)	9.54 ± 0.36^a^	9.56 ± 0.43^a^	9.32 ± 0.24^a^	9.24 ± 0.31^a^	9.24 ± 0.35^a^	9.19 ± 0.41^a^
Spread ratio	4.85 ± 0.32^a^	4.83 ± 0.21^a^	4.96 ± 0.16^a^	5.00 ± 0.23^a^	4.99 ± 0.31^a^	5.03 ± 0.25^a^
Moisture content(g/100 g)	1.87 ± 0.05^a^	1.94 ± 0.03^ac^	2.11 ± 0.07^b^	2.00 ± 0.03^c^	2.14 ± 0.04^b^	2.20 ± 0.05^bd^
Water-holding capacity(g/g)	0.31 ± 0.06^a^	0.41 ± 0.03^b^	0.44 ± 0.03^bc^	0.51 ± 0.02^de^	0.55 ± 0.04^de^	0.63 ± 0.03^f^
RDS/%	74.20 ± 2.14^ab^	68.87 ± 0.57^c^	66.23 ± 1.02^d^	62.84 ± 1.13^e^	59.81 ± 0.56^f^	57.77 ± 1.18^g^
SDS/%	22.45 ± 2.02^ab^	27.22 ± 1.15^c^	30.27 ± 1.45^d^	33.70 ± 1.12^e^	36.81 ± 0.33^f^	26.92 ± 0.95^c^
RS/%	3.35 ± 0.54^a^	3.41 ± 0.52^a^	3.51 ± 0.44^a^	3.46 ± 0.60^a^	3.38 ± 0.27^a^	3.37 ± 0.36^a^

Within a row, values (means ± SD, n = 3) marked with different superscript letters denote statistically significant differences (*P* < 0.05).

**Table 2:** Impact of *sophora japonica* flower powder addition (1%–5%) on the color, texture, physical properties, moisture content, water retention, and contents of RDS, SDS, and RS of biscuits.

Parameters	Control	*Sophora japonica* flower powder				
		1%	2%	3%	4%	5%
Color						
L^⁎^	49.19 ± 0.89^a^	48.67 ± 0.80^ab^	47.18 ± 0.52^c^	45.70 ± 0.43^d^	42.96 ± 0.59^f^	41.79 ± 0.55^g^
a^⁎^	8.16 ± 0.22^a^	7.50 ± 0.34^b^	7.44 ± 0.20^bc^	7.20 ± 0.14^cd^	7.00 ± 0.11^de^	6.80 ± 0.15^ef^
b^⁎^	27.17 ± 0.38^a^	26.27 ± 0.23^b^	25.77 ± 0.51^bc^	23.76 ± 0.39^d^	22.82 ± 0.67^e^	22.09 ± 0.63^fg^
ΔE	–	23.35 ± 0.50^a^	23.89 ± 0.41^a^	25.54 ± 0.65^b^	26.88 ± 0.37^c^	28.13 ± 0.33^d^
BI	88.35 ± 2.47^a^	85.03 ± 2.19^b^	86.54 ± 1.65^ab^	81.55 ± 2.23^d^	84.07 ± 1.61^bc^	83.56 ± 1.59^c^
Hardness (g)	24,360.35 ± 586.33^a^	21,542.06 ± 958.60^b^	17,792 ± 526.25^de^	19,985.83 ± 773.34^c^	17,076.39 ± 550.57^ef^	14,674.84 ± 501.61^g^
Crispness (g)	7376.29 ± 269.75^a^	7189.91 ± 75.89^ad^	6722.77 ± 104.75^b^	6951.78 ± 152.31^e^	6653.67 ± 112.58^b^	6089.45 ± 128.17^f^
Chewability (g∙cm)	8012.22 ± 308.66^a^	7980.49 ± 128.89^a^	7233.77 ± 203.37^cd^	7653.01 ± 300.34^b^	7267.25 ± 246.61^c^	6940.56 ± 151.54^d^
Diameter (mm)	46.30 ± 1.21^a^	46.16 ± 1.21^a^	46.18 ± 1.19^a^	46.34 ± 1.30^a^	46.22 ± 1.35^a^	46.17 ± 1.24^a^
Thickness (mm)	9.54 ± 0.36^a^	9.46 ± 0.31^a^	9.40 ± 0.34^a^	9.17 ± 0.37^a^	9.21 ± 0.26^a^	9.26 ± 0.39^a^
Spread ratio	4.85 ± 0.32^a^	4.87 ± 0.33^a^	4.91 ± 0.24^a^	5.05 ± 0.31^a^	5.02 ± 0.29^a^	4.99 ± 0.28^a^
Moisture content(g/100 g)	1.87 ± 0.05^a^	2.01 ± 0.05^c^	2.25 ± 0.06^de^	2.15 ± 0.04^b^	2.30 ± 0.06^e^	2.28 ± 0.08^de^
Water-holding capacity(g/g)	0.31 ± 0.06^a^	0.32 ± 0.04^a^	0.43 ± 0.03^b^	0.50 ± 0.02^cd^	0.57 ± 0.06^e^	0.55 ± 0.03^de^
RDS/%	74.20 ± 2.14^ab^	73.62 ± 1.66^a^	71.24 ± 1.09^b^	70.59 ± 0.74^b^	68.82 ± 1.03^c^	68.77 ± 0.91^c^
SDS/%	22.45 ± 2.02^ab^	22.52 ± 0.46^a^	23.65 ± 0.91^b^	23.73 ± 0.69^b^	24.14 ± 1.37^b^	26.92 ± 0.95^c^
RS/%	3.35 ± 0.54^a^	3.49 ± 0.45^a^	3.51 ± 0.35^a^	3.34 ± 0.23^a^	3.39 ± 0.34^a^	3.37 ± 0.36^a^

Within a row, values (means ± SD, n = 3) marked with different superscript letters denote statistically significant differences (*P* < 0.05).

**Fig. 3:**
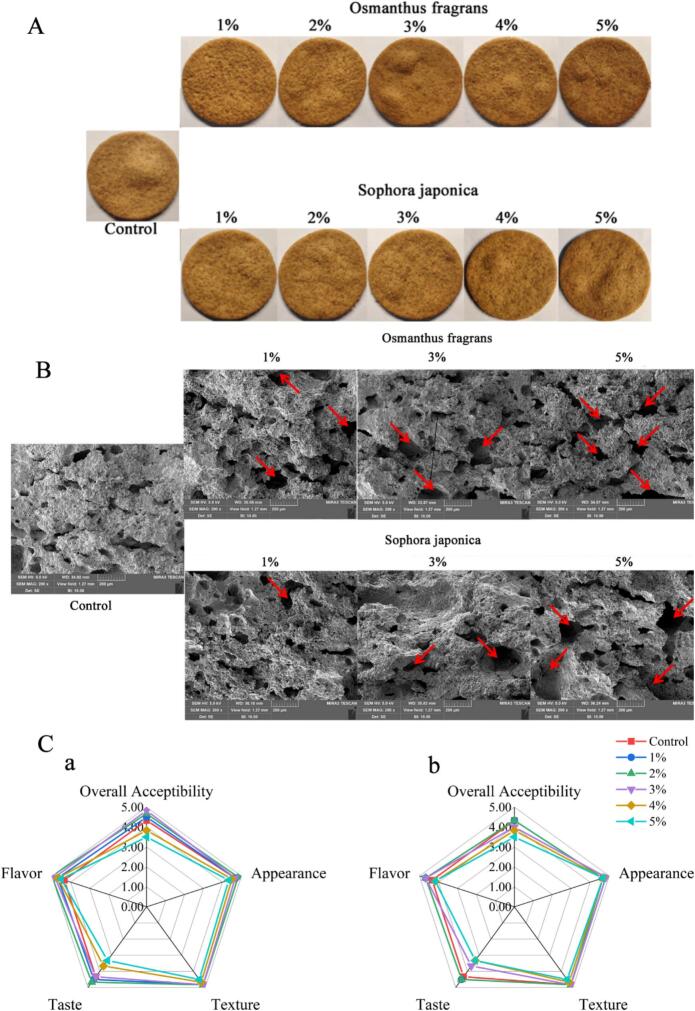
(A) Visual appearance of the biscuits with different addition levels (1%–5%) of osmanthus fragrans and *sophora japonica* flowers. (B) Surface microstructure of biscuits with different addition levels (1%–5%) of osmanthus fragrans and *sophora japonica* flowers, obtained from SEM imaging at different zones (200 ×).Arrows represent pores and voids. (C) Results of sensory evaluation of biscuits with different addition levels (1%–5%) of (a) *Osmanthus fragrans* and (b) *Sophora japonica* flower powders.

Biscuit color is closely associated with non-enzymatic browning pathways during baking, namely the Maillard reaction and caramelization. As shown in Fig. 3A, the color of biscuits darkened with increasing amounts of *Osmanthus fragrans* or *Sophora japonica* flower powder. Chromaticity analysis (Table 1, Table 2) indicated a decrease in lightness (L*)*,* redness (a*), and yellowness (b*), along with an increase in total color difference (ΔE), confirming that flower powder deepened biscuit color, consistent with reports for other flower such as dried blue pea (*Clitoria ternatea*) flower and male date palm flower powder-enriched biscuits (Antonyrasan et al., 2025; Karra et al., 2020). This effect can be attributed to: (1) amino acids and reducing sugars (e.g., glucose, fructose) in the flower powder enhancing Maillard reaction products; (2) caramelization of flower-derived sugars under high temperatures; and (3) natural pigments such as carotenoids in the flowers contributing to the final baked color. To further evaluate the effect of flower powder addition on biscuit browning, the browning index (BI) was calculated. Interestingly, the BI values of biscuits containing either flower powder were lower than the control at all addition levels, a trend opposite to the increase in ΔE (Tables 1 and 2). This divergence arises from the different principles behind the two metrics: ΔE quantifies the overall color shift and is largely driven by the marked decrease in L*, indicating general darkening. In contrast, BI specifically gauges non-enzymatic browning (Maillard reaction and caramelization) and is more sensitive to changes in a* and b*. The decline in a and b observed with flower-powder addition likely stems from both the intrinsic color of the floral pigments and the ability of phenolic compounds to interfere with Maillard-reaction intermediates. Consequently, the lower BI values suggest that the flower powders suppressed conventional non-enzymatic browning, while the rise in ΔE reflects the combined effect of the powders'own color and their modification of the biscuit matrix. These results support the use of edible flower powders as multifunctional ingredients that can enhance product appearance without promoting the formation of undesirable browning-related compounds.

Texture analysis (Table 1, Table 2) showed that flower powder significantly reduced hardness, crispness, and chewiness compared to the control, indicating improved crispness and tenderness. This aligns with findings from studies incorporating dietary fiber from kinnow peels and dried blue pea (*Clitoria ternatea*) flower powder (Antonyrasan et al., 2025; Kaur et al., 2023). Scanning electron microscopy (SEM; Fig. 3B) revealed microstructural changes underlying these textural modifications. Increasing flower powder addition introduced more pores and voids and reduced gluten network continuity. At the 5% addition level, the gluten structure exhibited tearing and loosening with enlarged gaps. This is likely due to components such as dietary fiber in the flower powder coating starch granules and physically impeding gluten network development (Raymundo et al., 2014), thereby reducing biscuit hardness.

Additionally, edible flower powder significantly increased both moisture content and water-holding capacity, with effects intensifying at higher incorporation levels (Table 1, Table 2). This is mainly due to the high hydration capacity of dietary fibers in the flowers (Jakubczyk et al., 2022). These fibers capture and immobilize water molecules through hydrogen bonding and other interactions, altering the distribution and state of water within the biscuit matrix (Jia et al., 2020). The water retention by dietary fibers, together with their disruption of the network structure, contributed to the reduction in hardness and overall textural modification. Similar trends were referred by other authors when adding male date palm flower powder and mango peel powder to biscuits formulations (Ajila et al., 2008; Karra et al., 2020).

The successful development of functional foods hinges on ingredients that provide added health benefits while preserving the sensory appeal of the final product. Sensory evaluation of the biscuits (Fig. 3C) confirmed that all formulations were generally acceptable, with mean scores above 3 on a 5-point scale. Biscuits containing 1%–3% (*w*/w) *Osmanthus fragrans* or 1%–2% (w/w) *Sophora japonica* flower powder scored comparably to or higher than the control in most sensory attributes. The highest overall acceptability was observed for *Osmanthus fragrans* at 1%–3%, likely due to its pleasant sweet and floral flavor. At higher substitution levels (≥4%), however, taste and overall acceptability declined, particularly for *Sophora japonica*, which may introduce undesirable bitter notes (Wei et al., 2018). These findings suggest that low to moderate incorporation of *Osmanthus fragrans* or *Sophora japonica* powder can enhance or maintain sensory quality while delivering potential functional benefits, supporting their further development as acceptable functional food ingredients.

### 3.3 Effects of flower powder addition on the contents of rapidly digestible starch (RDS), slowly digestible starch (SDS), and resistant starch (RS) in biscuits

Starch digestibility influences the nutritional function of food and its effect on postprandial blood glucose. In this study, biscuits were prepared with 1%–5% *Osmanthus fragrans* or *Sophora japonica* flower powder, and the contents of RDS, SDS, and RS were analyzed using an in vitro simulated digestion model. As shown in Table 1, Table 2, compared with the control, the addition of *Osmanthus fragrans* flower powder significantly altered the starch digestive fractions in biscuits: RDS content decreased, while SDS content increased, with no significant change in RS. At the 5% addition level, the RDS content was 57.77%, representing a 22.14% reduction relative to the control, while SDS increased to 39.11%, a 16.66% rise. The shift is likely associated with changes in the physical state of starch within the biscuit matrix induced by the flower powder. Active components in *Osmanthus fragrans* flower powder, such as polyphenols and dietary fiber, may form hydrogen bonds with starch molecules or create physical barriers, thereby slowing the enzymatic hydrolysis rate and facilitating the conversion of RDS to SDS (Mackie et al., 2016).

A similar trend in RDS, SDS, and RS contents was observed in biscuits containing *sophora japonica* flower powder. However, at equivalent addition levels, *Osmanthus fragrans* flower biscuits exhibited lower RDS and higher SDS contents than those with *Sophora japonica* flower. This difference may be due to the higher intrinsic sugar content (e.g., glucose and fructose) in *Sophora japonica* flowers, which could compete for water during baking and weaken the interaction between functional components (e.g., fiber) and starch, thereby moderating its impact on starch digestibility.

### 3.4 Effects of flower powder addition on starch hydrolysis rate in biscuits

**Fig. 4:**
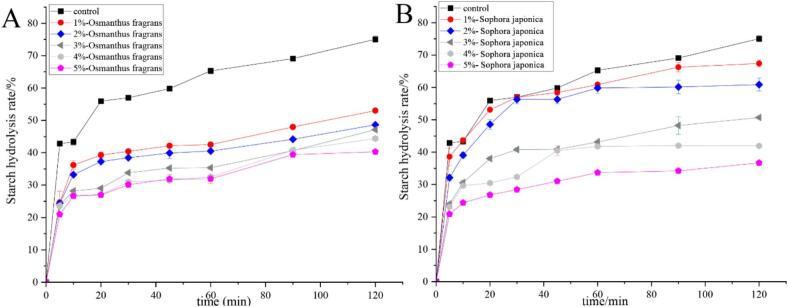
The starch digestion curves of biscuits up to 120 min with different addition levels (1%–5%) of osmanthus fragrans and *sophora japonica* flowers.

The rate of enzymatic starch hydrolysis during digestion reflects the rate of food digestion. The slower the hydrolysis rate, the slower the starch digestion rate (Wang et al., 2022). Fig. 4 shows the starch digestion curves of biscuits containing different types and levels of flower powder over 120 min. As shown in Fig. 4A, biscuits with *Osmanthus fragrans* flower powder exhibited a rapid increase in hydrolysis within the first 20 min, followed by a gradual rise up to 120 min. Throughout this period, the hydrolysis rate remained lower than that of the control. This result aligns with the observed reduction in RDS and increase in SDS in *Osmanthus fragrans* flower-enriched biscuits (Table 1, Table 2). A similar hydrolysis trend was observed for *sophora japonica* flower biscuits (Fig. 4B). However, at each time point, their hydrolysis rates were higher than those of *Osmanthus fragrans* flower biscuits, though still lower than the control. This is consistent with the less pronounced changes in RDS and SDS content shown in Table 1, Table 2. These hydrolysis profiles confirm that *Osmanthus fragrans* flower powder significantly reduces RDS and increases SDS in biscuits, while *Sophora japonica* flower exerts a similar but weaker effect. The shift from RDS to SDS by *Osmanthus fragrans* flower powder is similar to the effect of chickpea flour, a source rich in dietary fiber and polyphenols, which elevated SDS levels by 15–20% in biscuits (Lu et al., 2022).

### 3.5 Effect of aqueous extracts from biscuits with flower powder on estimated glycemic index (eGI)

**Fig. 5:**
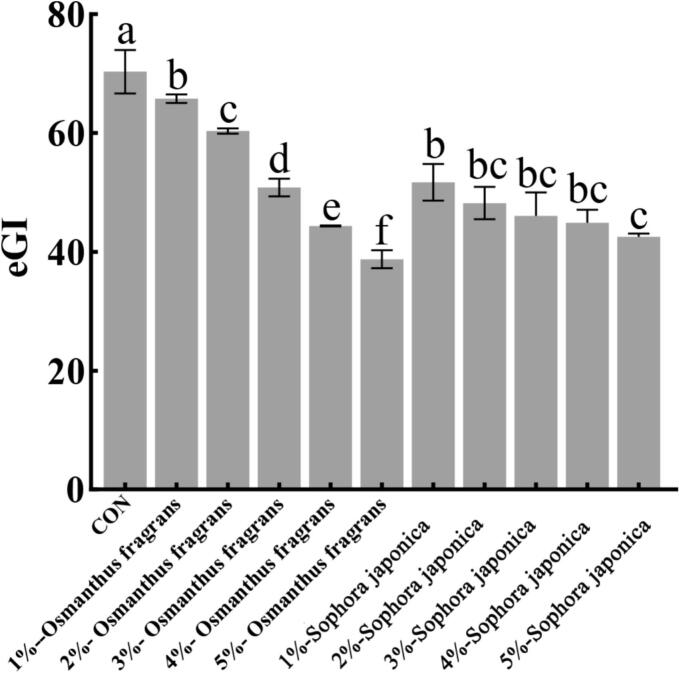
The eGI of biscuits with different addition levels (1%–5%) of osmanthus fragrans and *sophora japonica* flowers.

The estimated glycemic index (eGI) provides an efficient in vitro predictor of a food's potential to raise blood glucose levels. The addition of *Osmanthus fragrans* or *sophora japonica* flower powder significantly reduced the eGI of the biscuits (Fig. 5), suggesting a potential function in delaying starch digestion and modulating postprandial glycemic response. This finding is consistent with and mutually supportive of the previously observed results, namely the reduction in RDS and the increase in SDS, collectively supporting the starch-modulating effect of the flower powders.

### 3.6 Effects of aqueous extracts from biscuits with flower powder on the starch-digesting enzymes activity

**Fig. 6:**
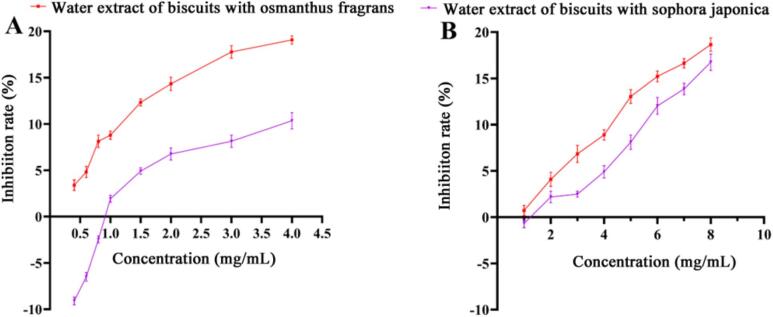
The effects of aqueous extracts from biscuits containing osmanthus fragrans or *sophora japonica* flowers on the activities of (A) α-amylase and (B) α-glucosidase.

α-Amylase and α-glucosidase are key enzymes in starch digestion. α-Amylase cleaves internal α-(1 → 4) glycosidic bonds in starch to produce maltose and oligosaccharides, which are further hydrolyzed into absorbable glucose by α-glucosidase (Papoutsis et al., 2021). This study examined the effects of aqueous extracts from biscuits containing *Osmanthus fragrans* or *Sophora japonica* flower powders on the activities of these enzymes. As shown in Fig. 6A, the aqueous extract of *Osmanthus fragrans* flower biscuits significantly inhibited α-amylase in a concentration-dependent manner.In contrast, the extract from *sophora japonica* flower biscuits slightly promoted α-amylase activity at low concentrations (< 0.8 mg/mL) but shifted to concentration-dependent inhibition at higher concentrations (0.8–2.0 mg/mL). At equivalent concentrations, the inhibitory effect of *Osmanthus fragrans* flower biscuit extract was significantly stronger than that of the *sophora japonica* flower biscuit extract. Both extracts also inhibited α-glucosidase activity in a concentration-dependent manner (Fig. 6B), with *Osmanthus fragrans* flower biscuit extract again showing greater inhibition. The stronger inhibitory effect of *Osmanthus fragrans* flower biscuit extracts on α-amylase and α-glucosidase may be attributed to its higher content of verbascoside (Table S1). Verbascoside inhibits α-amylase by binding to its active site through hydrogen bonding and hydrophobic interactions, which subsequently induces conformational changes in the enzyme's secondary structure (Li et al., 2025). Similarly, it suppresses α-glucosidase activity by forming stable hydrogen bonds with multiple key residues in the enzyme's active site, thereby disrupting its catalytic function (Ritter Ruas et al., 2023). These findings suggest that consumption of *Osmanthus fragrans*-enriched biscuits may slow the conversion of starch to glucose in vivo, highlighting its potential for moderating postprandial glycemic responses.

## 4 Conclusion

This study demonstrates that incorporating selected edible flower powders, especially from *Osmanthus fragrans*, effectively enhances both the safety and health-related properties of biscuits. *Osmanthus fragrans* flower powder significantly and dose-dependently inhibited the formation of Maillard reaction-derived hazards, including AA, 5-HMF, and AGEs. The incorporation of flower powders also improved biscuit texture, modified the microstructure, and increased water-holding capacity. Moreover, a favorable shift in starch digestibility was observed, marked by reduced rapidly digestible starch and increased slowly digestible starch, accompanied by a lower estimated glycemic index. These beneficial effects are potentially attributed to two main mechanisms: inhibition of activities of digestive enzymes (α-amylase and α-glucosidase) by polyphenols, and delayed starch hydrolysis due to dietary fiber interfering with the starch-gluten matrix. Overall, our findings support the use of certain edible flowers, such as *Osmanthus fragrans* flower, as natural dual-functional ingredients for developing safer and metabolically favorable baked products. Limitations of this study include the use of only in vitro digestion models. Future studies should verify the regulatory effects on starch digestion using in vivo animal model and explore the stability of flower-derived bioactive compounds during baking.

## CRediT authorship contribution statement

**Yueliang Zhao:** Writing – original draft, Methodology, Investigation, Funding acquisition, Conceptualization. **Xiangru Zhao:** Methodology, Investigation. **Daming Fan:** Conceptualization. **Mingfu Wang:** Writing – review & editing. **Hui Wang:** Writing – review & editing, Supervision, Funding acquisition, Conceptualization.

## Declaration of competing interest

The authors declare that they have no known competing financial interests or personal relationships that could have appeared to influence the work reported in this paper.

## Data Availability

Data will be made available on request.
