# Supplementary material for: Effects of Osmanthus fragrans and Sophora japonica flower powders addition on hazardous compound formation, quality characteristics, and starch digestion in baked biscuits
Source: Food Chem X. 2026 Apr 25;36:103915. doi: 10.1016/j.fochx.2026.103915 (PMC13138065; doi:10.1016/j.fochx.2026.103915)
Supplement: Supplementary file 1 — Supplementary material [file mmc1.docx]

**Supplementary Materials For:**

**Effects of Osmanthus fragrans and Sophora japonica flower powders addition on hazardous compound formation, quality characteristics, and starch digestion in baked biscuits**

Yueliang Zhao^a,^*, Xiangru Zhao^b^, Daming Fan^c^, Mingfu Wang^d^, Hui Wang^a,^*

^a^ School of Public Health, Shanghai Jiao Tong University School of Medicine, Shanghai, 200025, China

^b^ College of Food Science and Technology, Shanghai Ocean University, Shanghai, 201306, China

^c^ School of Food Science and Technology, Jiangnan University, Wuxi, China

^d^ Shenzhen Key Laboratory of Food Nutrition and Health, College of Chemistry and Environmental Engineering, Shenzhen University, Shenzhen, 518060, PR China

To whom correspondence should be addressed:

*Prof. Hui Wang, School of Public Health, Shanghai Jiao Tong University School of Medicine, E-mail: huiwang@shsmu.edu.cn

*Prof. Yueliang Zhao, School of Public Health, Shanghai Jiao Tong University School of Medicine, E-mail: ylzhao1@sjtu.edu.cn

Table S1 The phenolic profile of Osmanthus fragrans and Sophora japonica flower. Phenolic compounds were extracted from each edible flower powder (10 g) using 100 mL of water and anhydrous ethanol, respectively. For each solvent, the sample was homogenized, ultrasonicated for 30 min, and filtered; the residue was re‑extracted twice and the combined extracts were concentrated to dryness by rotary evaporation. The phenolic profile, including chlorogenic, syringic, caffeic, protocatechuic, and p-hydroxybenzoic acids, as well as quercetin, rutin, and verbascoside, was analyzed by HPLC. Extracts were reconstituted in ultrapure water or methanol (1 mg/mL), filtered (0.22 μm), and separated on a YMC‑Pack ODS C18 column (250 mm × 4.6 mm, 5 μm) with a gradient of 0.1% formic acid (A) and acetonitrile (B) at 1 mL/min. The gradient program was: 0–1 min, 90% A; 13 min, 72% A; 16 min, 0% A; 17–18 min, 0% A; 18–22 min, 90% A. The injection volume was 10 μL. Verbascoside was detected at 330 nm and other phenolics at 280 nm. Identification was based on retention time and UV spectra, and quantification was performed using external calibration. All analyses were conducted in triplicate.

|  | Osmanthus fragrans | Sophora japonica flower | Osmanthus fragrans | Sophora japonica flower |
| --- | --- | --- | --- | --- |
|  | Aqueous extracts | Aqueous extracts | Ethanol extracts | Ethanol extracts |
| Syringic acid | 3.18±0.12 | 3.58±0.13 | 3.02±0.08 | 3.87±0.14 |
| Caffeic acid | 3.38±0.15 | 3.16±0.14 | 3.49±0.12 | 3.14±0.12 |
| Chlorogenic acid | 16.06±1.05 | 17.20±1.42 | 14.36±1.15 | 17.79±1.14 |
| Protocatechuic acid | 5.54±0.42 | 7.69±0.28 | 5.73±0.31 | 7.92±0.34 |
| p-hydroxybenzoic acid | 6.74±0.51 | 5.21±0.18 | 6.10±0.34 | 4.83±0.19 |
| Quercetin | N.D. | N.D. | 5.23±0.04 | 44.48±1.76 |
| Rutin | N.D. | 7.40±0.24 | 33.72±2.47 | 167.94±14.52 |
| Verbascoside | 414.46±27.65^a^ | N.D. | 452.32±28.34 | N.D. |

N.D., not detectable. The results are expressed as mg/L.
